# The role of diabetes in cardiomyopathies of different etiologies—Characteristics and 1-year follow-up results of the EVITA-HF registry

**DOI:** 10.1371/journal.pone.0234260

**Published:** 2020-06-11

**Authors:** Christine Meindl, Matthias Hochadel, Lutz Frankenstein, Oliver Bruder, Matthias Pauschinger, Rainer Hambrecht, Wolfgang von Scheidt, Otmar Pfister, Andreas Hartmann, Lars S. Maier, Jochen Senges, Bernhard Unsöld

**Affiliations:** 1 Department of Internal Medicine II, University Hospital Regensburg, Regensburg, Germany; 2 Stiftung Institut für Herzinfarktforschung Ludwigshafen, Ludwigshafen, Germany; 3 Department of Internal Medicine III, University Hospital Heidelberg, Heidelberg, Germany; 4 Department of Cardiology and Angiology, Elisabeth-Hospital Essen, Essen, Germany; 5 Department of Internal Medicine 8, Nürnberg Hospital, Nürnberg, Germany; 6 Department of Internal Medicine II, Hospital Links der Weser, Bremen, Germany; 7 Department of Internal Medicine I, University Hospital Augsburg, Augsburg, Germany; 8 Department of Cardiology, University Hospital Basel, Basel, Switzerland; 9 Department of Cardiology and Internal Intensive Care, St. Georg Hospital Leipzig, Leipzig, Germany; Erasmus Medical Center, NETHERLANDS

## Abstract

**Background:**

Type 2 diabetes is a major risk factor for cardiovascular diseases, e.g. coronary artery disease (CAD). But it has also been shown that diabetes can cause heart failure independently of ischemic heart disease (IHD) by causing diabetic cardiomyopathy. In contrast to diabetes and IHD, limited data exist regarding patients with diabetes and dilated cardiomyopathy (DCM).

**Methods:**

EVIdence based TreAtment in Heart Failure (EVITA-HF) comprises web-based case report data on demography, diagnostic measures, adverse events and 1-year follow-up of patients hospitalized for chronic heart failure and an ejection fraction ≤40%. In the present study we focused on the results of patients with diabetes and heart failure.

**Results:**

Between February 2009 and November 2015, 4101 patients with chronic heart failure were included in 16 tertiary care centers in Germany. The mortality in patients with diabetes and DCM (n = 323) was more than double (15.2%) than that of DCM patients without diabetes (6.5%, p<0.001, n = 885). In contrast the mortality rate of patients with **IHD** was not influenced by the presence of diabetes (17.6% in patients with **IHD** and diabetes n = 945, vs. 14.7% in patients with **IHD** and no diabetes, n = 1236, p = 0.061). The results also remained stable after performing a multivariable analysis (unadjusted p-value for interaction = 0.002, adjusted p = 0.046).

**Conclusion:**

The influence of diabetes on the mortality rate is only significant in patients with DCM not in patients with CAD. Therefore, the underlying mechanisms of this effect should be studied in greater detail to improve patient care and outcome.

## Introduction

Type 2 diabetes is a major risk factor for cardiovascular diseases [1]. But already more than 40 years ago Rubler et al. described the development of heart failure in patients with diabetes independently of well-established risk factors for heart failure such as hypertension or ischemic heart disease [2]. Today, there is increasing evidence that diabetes can cause heart failure independently of ischemic heart disease by causing a diabetic cardiomyopathy [3]. The definition of diabetic cardiomyopathy is a ventricular dysfunction in the absence of relevant coronary artery disease and hypertension in patients with diabetes [4]. Epidemiological studies have shown that the incidence of heart failure is up to 4-fold higher in people with diabetes compared to those without diabetes [5, 6].

Despite this fact in patients with heart failure, irrespective of it’s etiology, diabetes as comorbidity unfavorably affects prognosis [7]. Concerning the etiology of diabetic cardiomyopathy several mechanisms have been discussed. For example, it is argued that myocardial inflammation is a possible pathophysiologic process contributing to cardiac hypertrophy, fibrosis and dysfunction in the context of heart disease [8–10]. There is evidence that myocardial inflammation is a contributor to the development of diabetic cardiomyopathy [8–11].

Several pathological insults may cause myocardial inflammation, which at first represents an adaptive mechanism against stress, but which can lead to maladaptive processes if the stress persists [8–10].

The impact of myocardial inflammation on cardiac remodeling and heart failure development seems to be important for human diseases. In patients with heart failure higher circulating cytokine levels were measured and they were directly correlated with the severity of the disease and with a poor prognosis [12–14].

Results of the Framingham Heart Study showed that patients without a prior acute myocardial infarction who had higher baseline levels of TNF-α, IL-6 and C-reactive proteins (CRP) had a significantly higher long-term risk of developing heart failure, independently of the occurrence of an acute myocardial infarction [15].

A prior study even showed a worse hemodynamic status in patients with diabetes-related cardiomyopathy with a dilated HFrEF phenotype compared to other dilated cardiomyopathy patients having a lower LVEF and a higher myocardial stiffness modulus [16].

It is hypothesized that the detrimental effect of diabetes mellitus on the myocardium is associated with metabolic abnormalities such as advanced glycosylation end products (AGEs) deposition, lipotoxicity and microvascular rarefication [17].

As we focus on the effect of heart failure therapies, subgroup analyses of diabetic populations have demonstrated that despite the increased risk of morbidity and mortality [18], patients with diabetes can also benefit more from efficacious therapies [19–21]. That is why patients with diabetes should be treated the same way as patients without diabetes always keeping in mind comorbidities such as renal dysfunction and hyperkalemia [22].

The EVIdence based TreAtment in Heart Failure (EVITA-HF) registry has already been described elsewhere [23]. EVITA-HF evaluates demography, comorbidities, diagnostic and therapeutic interventions, quality of life and adverse events in patients with chronic heart failure and an ejection fraction of 40% or lower. The data derive from an index hospital stay or an index outpatient visit, and a 1-year follow-up of these patients [23]. In the present study we focused on the results of patients with diabetes and heart failure.

## Materials and methods

EVITA-HF is a registry of heart failure patients from 16 European tertiary care centers which offer the whole spectrum of diagnostic and treatment modalities in heart failure. Patients were hospitalized in one of the 16 participating hospitals and had to be included consecutively. As already described by von Scheidt et al. [23] inclusion criteria were chronic heart failure ≥ 3 months and a documented ejection fraction ≤ 40%. Exclusion criteria consisted of age younger than 18 years or missing consent of the patient. Case report data was collected using a web-based electronic case report form (eCRF). Data management was performed at the Institut für Herzinfarktforschung Ludwigshafen at the University of Heidelberg, Germany [23].

The registry was approved by the following ethics committees: State medical association of Rhineland-Palatinate, Germany; University of Bonn, Germany; University of Rostock, Germany; State medical association of Bavaria, Germany; University of Basel, Switzerland; Canton Aargau, Switzerland. Informed written consent was obtained from every patient. The registry was supported by unrestricted grants from Medtronic, Novartis and Sanofi Aventis [23].

Baseline information concerning demographics, medical history, clinical evaluation and diagnostics as well as pharmacological and non-pharmacological treatment, quality of life and adverse events during index hospitalization were gathered by eCRF. In some participating centers a representative one-year follow-up was performed by phone calls and/or contact by the center or general practitioner [23].

Follow-up data consisted of vital status, adverse events and interventions since index discharge and current health status, pharmacological treatment and quality of life. 1-year follow-up was defined as status obtained between 300 and 450 days after index discharge [23]. All cause mortality was used for mortality analyses. The EVITA-HF trial started in January 2009 and included 4101 patients until November 2015. The final analysis of the data was performed in February 2017.

### Statistical analysis

The patient population was described by absolute numbers and percentages with respect to categorical variables and by medians with quartiles for continuous variables. The distribution of dichotomous variables was compared between patient groups by Pearson Chi-square test, and odds ratios with 95%-confidence intervals were calculated. The Mann-Whitney test was used for the comparison of metrical or ordinal variables.

One-year survival and event-free survival after index discharge were analyzed using the product-limit method and the log-rank test. The results are demonstrated in Kaplan-Meier curves for patients with vs. without diabetes in the total cohort and in the subgroups with **IHD** and with DCM but without CAD. Corresponding hazard ratios were calculated in Cox regression models, unadjusted as well as adjusted for the clinically relevant risk factors age, sex, LVEF ≤ 30%, NYHA III/IV on admission, chronic kidney disease and atrial fibrillation. The interaction between the two subgroups was assessed by the Wald test.

All tests performed were two-sided and a p-value ≤ 0.05 was considered statistically significant. The computations were performed using the SAS system (release 9.4, SAS Institute, Inc., Cary, North Carolina).

## Results and discussion

### Baseline characteristics of the EVITA-HF cohort and the diabetes subgroup

Between February 2009 and November 2015, 4101 patients with chronic heart failure were included in 16 European tertiary care centers. The study cohort consisted of 1489 patients (36.3%) with diabetes and 2612 patients (63.7%) without diabetes. In Table 1, patient characteristics of the EVITA-HF patients and the diabetic subgroup are shown. Patients without diabetes were younger compared to diabetics (68 vs. 70 years, p<0.001). Patients with diabetes suffered more often from ischemic heart disease than non-diabetics (63.6 vs. 47.4%, p<0.001) and more often had a history of myocardial infarction (44.7% vs. 33.6%, p<0.001) as well as of revascularisation (53.8% vs. 37.3%, p<0.001). Patients with diabetes had been hospitalized more often because of heart failure (68.4% vs. 60.1%, p = 0.001) than non-diabetics. Dilated cardiomyopathy was present in 25.3% of diabetics and 37.5% of non-diabetics (<0.001).

**Table 1:** Baseline characteristics of the EVITA-HF cohort and the diabetes subgroup.

	Diabetes	No diabetes	p-value	Odds Ratio (95%-confidence interval)
n—(%)	1489 (36.3)	2612 (63.7)		
Age—yrs ‡	70 (62;76)	68 (55;76)	<0.001	
Male gender—(%) n	77.8 (1158/1489)	75.7 (1977/2612)	0.13	1.12 (0.97–1.31)
Ischemic heart disease—% (n/total n)	63.6 (945/1487)	47.4 (1236/2606)	<0.001	1.93 (1.70–2.20)
Dilated CMP—% (n/total n)	25.3 (376/1487)	37.5 (976/2606)	<0.001	0.57 (0.49–0.65)
Myocardial infarction—% (n/total n)	44.7 (666/1489)	33.6 (876/2608)	<0.001	1.60 (1.40–1.82)
Revascularization (PCI and/or CABG)—% (n/total n)	53.8 (764/1421)	37.3 (917/2457)	<0.001	1.95 (1.71–2.23)
PCI—% (n/total n)	39.1 (556/1421)	28.6 (703/2457)	<0.001	1.60 (1.40–1.84)
CABG—% (n/total n)	26.0 (369/1421)	15.6 (383/2457)	<0.001	1.90 (1.62–2.23)
Valve surgery/intervention—% (n/total n)	5.8 (83/1420)	6.6 (162/2456)	0.35	0.88 (0.67–1.16)
Atrial fibrillation—% (n/total n)	38.3 (571/1489)	35.2 (920/2610)	0.047	1.14 (1.00–1.30)
Hypertension—% (n/total n)	79.0 (1176/1489)	64.9 (1695/2612)	<0.001	2.03 (1.75–2.36)
Stroke—% (n/total n)	8.3 (124/1488)	7.5 (196/2609)	0.35	1.12 (0.89–1.41)
Peripheral artery disease—% (n/total n)	14.9 (221/1488)	8.0 (208/2607)	<0.001	2.01 (1.65–2.46)
Chronic kidney disease—% (n/total n)	40.8 (607/1489)	26.8 (699/2611)	<0.001	1.88 (1.64–2.15)
Previously hospitalized for heart failure—% (n/total n)	68.4 (569/832)	60.1 (861/1433)	<0.001	1.44 (1.20–1.72)
Implanted device (ICD, CRT-D, CRT-P, PM)—% (n/total n)	38.4 (571/1486)	34.5 (899/2609)	0.011	1.19 (1.04–1.35)

^‡^ median (quartiles), yrs years, CMP cardiomyopathy, PCI percutaneous coronary intervention, CABG coronary artery bypass graft, PM pacemaker, ICD implantable cardioverter-defibrillator, CRT cardiac resynchronization therapy.

Atrial fibrillation (38.3% vs. 35.2%, p = 0.047) and arterial hypertension (79.0% vs. 64.9%, p<0.001) were diagnosed more often in the diabetic vs. non-diabetic group. Renal insufficiency was found more often in diabetic patients than in non-diabetics (40.8% vs. 26.8%, p<0.001). Concerning the device status 24% of diabetics had had an ICD-implantation and 8.1% carried a cardiac resynchronization therapy defibrillator (CRT-D) compared to 21.1% (p = 0.033) and 7.5% with an ICD or CRT-D respectively in the non-diabetes group (p = 0.48) (S1 Table).

In Table 2 clinical and technical findings of patients with diabetes and without diabetes at index presentation are depicted. No differences occurred concerning left ventricular (LV) function (median LVEF 30% in both groups). In contrast differences were found in the values of body mass index (BMI) (28.4 kg/m^2^ in diabetics vs. 26.2 kg/m^2^ in non-diabetics, p<0.001) and also the functional status (NYHA class IV 14.9% of subjects with diabetes vs. 13.5% in non-diabetics, p<0.001). Using the Minnesota Living with Heart Failure questionnaire (MLWHFQ) patients with diabetes had a poorer quality of life (MLWHFQ Score 40 vs. 32, <0.001) than patients without diabetes.

**Table 2:** Clinical and technical findings at index presentation.

	Diabetes	No diabetes	p-value
n, (%)	1489 (36.3)	2612 (63.7)	
In-patient stay, % (n)	76.2 (1135)	68.6 (1792)	<0.001
Outpatient clinic, % (n)	23.8 (354)	31.4 (820)	<0.001
BMI (kg/m^2^) ‡	28.4	26.2	<0.001
Systolic blood pressure—mmHg ‡	120 (110;140)	120 (110;135)	<0.001
Diastolic blood pressure—mmHg ‡	70 (64;80)	70 (65;80)	0.39
NYHA functional class:			<0.001
NYHA class I—% (n/total n)	7.5 (111/1488)	13.0 (340/2606)	
NYHA class II—% (n/total n)	31.3 (465/1488)	33.1 (863/2606)	
NYHA class III—% (n/total n)	46.4 (690/1488)	40.3 (1051/2606)	
NYHA class IV—% (n/total n)	14.9 (222/1488)	13.5 (352/2606)	
LVEF—% ‡	30 (23;35)	30 (23;35)	0.71
LVEF ≤ 30%—% (n/total n)	62.8 (935/1489)	60.7 (1586/2612)	0.19
Quality of life—MLWHFQ Score ‡*	40 (25;57)	32 (17;50)	<0.001

^‡^ median (quartiles), BMI body mass index, NYHA New York Heart Association, LVEF left ventricular ejection fraction,

* documented in 36% of patients only due to later introduction in June 2011.

In S1 Table medication and device status at admission and discharge are shown. Comparing both groups significant differences were observed in the prescription of diuretics (80.8% in diabetics vs. 65.1% in non-diabetics, p<0.001), digitalis (23.6% in diabetics vs. 14.6% in non-diabetics, p<0.001) and statins (64.6% in diabetics vs. 48.9% in non-diabetics, p<0.001).

As already described previously [23] the number of patients treated with ACE inhibitors (ACEI)/angiotensin receptor blockers (ARB), ß-blockers, mineral corticosteroid receptor antagonists (MRA) and diuretics increased in both groups from admission to discharge (S1 Table). Similar results were found concerning implanted devices especially ICDs and CRT-Ds (S1 Table).

**Fig 1:**
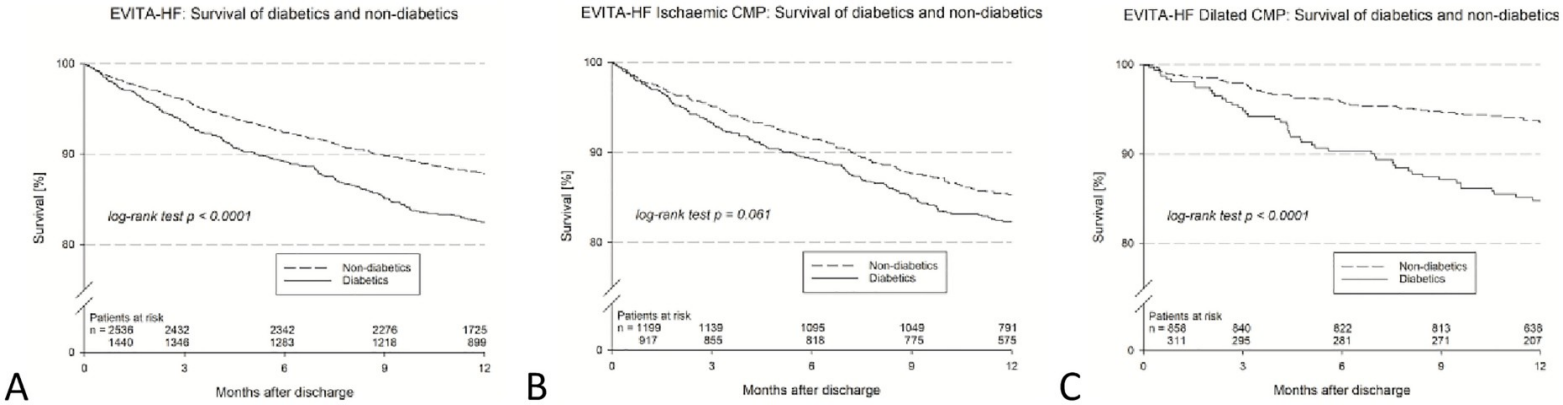
A) Survival of diabetics and non-diabetics. B) Survival of diabetics and non-diabetics in patients with IHD. C) Survival of diabetics and non-diabetics in patients with DCM.

One year follow-up data was available in 98.4% of patients with diabetes and 98.3% in patients without diabetes (Table 3). 1-year mortality (all cause) was 17.5% in diabetics and 12.3% in non-diabetics (p<0.001 log-rank test, Table 3 & Fig 1A). Major adverse cardiac and cerebrovascular events (MACCE) occurred in 19.0% of diabetics compared to 13.8% in non-diabetics (p<0.001 log-rank test). As already noted at baseline the functional status remained worse in patients with diabetes (37.4% of patients in NYHA class III/IV) than in non-diabetics (28.0% NYHA status III/IV, p<0.001). In S2 Table the medication of patients with and without diabetes at one year follow-up is depicted. Statistically significant differences were only observed in the use of diuretics and digitalis.

**Table 3:** Events and findings in patients with complete one-year follow-up (FU).

	Diabetes	No diabetes	p-value	Odds Ratio (95%-confidence interval)
FU available %, (n)	98.4 (1440/1464)	98.3 (2536/2579)	0.95	1.02 (0.61–1.68)
FU duration, months, median (IQR)	12.5 (12.0;13.9)	12.5 (12.0; 13.7)	0.81	
1-year all cause mortality‡	17.5%	12.3%	<0.001	1.48 (1.25–1.75)
Death or rehospitalization‡	52.6%	44.1%	<0.001	1.29 (1.17–1.43)
Death, myocardial infarction or stroke (MACCE) ‡	19.0%	13.8%	<0.001	1.42 (1.21–1.67)
1-year status available, n (%)*	928 (34.5)	1760 (65.5)		
NYHA status I/II, % (n)	62.6 (453/724)	72.0 (1019/1415)	<0.001	0.65 (0.54–0.79)
NYHA status III/IV, % (n)	37.4 (271/724)	28.0 (396/1415)	<0.001	1.54 (1.27–1.86)
Atrial fibrillation, % (n)	19.3 (141/732)	18.3 (258/1419)	0.59	
Implanted device, % (n)	58.8 (545/927)	51.3 (902/1760)	<0.001	1.36 (1.16–1.59)
ICD, % (n)	34.8 (322/925)	30.9 (541/1751)	0.039	1.19 (1.01–1.41)
CRT-D, % (n)	17.1 (158/925)	14.2 (249/1751)	0.05	1.24 (1.00–1.54)
PCI, % (n)	2.2 (18/816)	2.0 (31/1562)	0.72	1.11 (0.62–2.00)
CABG, % (n)	1.0 (8/816)	0.6 (10/1562)	0.36	1.54 (0.60–3.91)

FU follow-up, IQR interquartile range, MACCE major adverse cardiac and cerebrovascular events, NYHA New York Heart Association, ICD implantable cardioverter-defibrillator, CRT cardiac resynchronization therapy, PCI percutaneous coronary intervention, CABG coronary artery bypass graft.

^‡^ Kaplan-Meier estimates at 366 days after index discharge, p-values of the log-rank test, and hazard ratios are presented.

* Documentation of follow-up interview with survivors between 300 and 450 days after index discharge.

* adjusted for age, sex, LVEF</ = 30%, NYHA III+ on admission, chronic kidney disease and atrial fibrillation.

### Patient characteristics of the DCM cohort (with and without diabetes) compared to the IHD group (with and without diabetes)

S3 Table shows demographics and findings in patients with DCM and diabetes (n = 323), DCM and no diabetes (n = 885) as well as in patients with IHD and diabetes (n = 945) and patients with IHD but without diabetes (n = 1236).

Comorbidities such as arterial hypertension and chronic kidney disease were found more often in diabetics in the DCM and also in the IHD cohort (S3 Table, supporting information). Diabetics were in a statistically significant poorer functional status (NYHA class III+) compared to non-diabetics in the DCM but also in the IHD group. While LVEF was not different between the groups, the need for diuretic medication was significantly higher in diabetics in the DCM as well as in the IHD cohort.

In S4 Table the results of the one year follow-up data of the DCM cohort (with and without diabetes) and the IHD group (with and without diabetes) are depicted.

**Fig 2:**
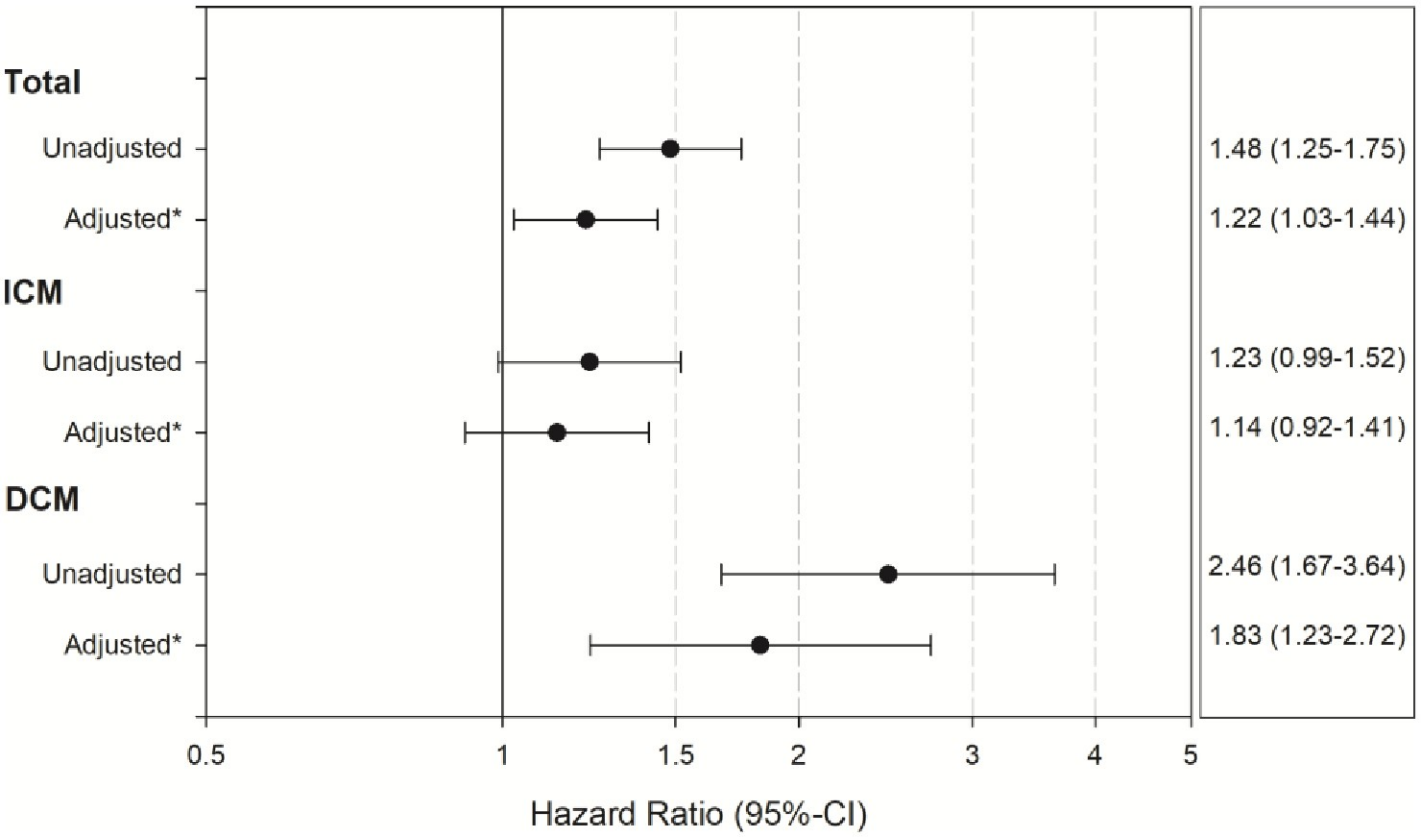
Results of the multivariate analysis for the influence of diabetes on mortality.

Interestingly the all cause mortality in patients with diabetes and DCM (15.2%) was more than double than that of DCM patients without diabetes (6.5%, p <0.001, S4 Table and Fig 1C). In contrast the all cause mortality rate of patients with IHD was not influenced by the presence of diabetes (17.6% in patients with IHD and diabetes vs. 14.7% in patients with IHD and no diabetes, S4 Table, Fig 1B). The results also remained stable after performing a multivariable analysis (unadjusted p-value for interaction = 0.002, adjusted p = 0.046, Fig 2).

## Discussion

One of our major findings was that the mortality in patients with diabetes and DCM was significantly higher (15.2%) than in patients with DCM and no diabetes (6.5%). The mortality rate of patients with DCM and diabetes was similar to that of patients with IHD (17.6% in patients with IHD and diabetes vs. 14.7% in patients with IHD and no diabetes).

Our results emphasize the fact that the combination of heart failure and diabetes mellitus influences clinical management and prognosis. It is known that diabetes mellitus is associated with worse clinical status and increased all-cause and cardiovascular mortality in patients with heart failure and reduced ejection fraction (HFrEF) as well as in patients with preserved ejection fraction (HFpEF) [24]. Our finding that the negative effect of diabetes on prognosis is more distinct in patients with dilated cardiomyopathy compared to patients with ischemic heart disease corroborates the hypothesis of a possible direct detrimental effect of diabetes mellitus on the myocardium [17].

Several studies as the Reykjavik [25] and the England study [26] revealed a much higher prevalence of type 2 diabetes in patients with heart failure (Reykjavik study 12%, England study 24%) than without heart failure in the general population (Reykjavik study 3%, England study 3%) [27].

In recent clinical trials of patients with chronic heart failure e.g. PARADIGM-HF [28] and SHIFT [29] the prevalence of type 2 diabetes was around 30% (35% in the PARADIGM-HF vs. 30% in the SHIFT trial). These results are comparable to the prevalence of type 2 diabetes in the EVITA-HF study population (36%).

Our data also confirm the results of prior studies showing that patients with type 2 diabetes and both HFrEF [24, 30–32] and HFpEF [24] are in a worse NYHA class and are suffering from more HF-related symptoms than patients without diabetes, despite having similar ejection fraction [31, 32].

Several clinical trials could corroborate a similar effectiveness of pharmacological and device therapy in patients with and without diabetes [27].

In the present analysis significant differences concerning medical treatment between diabetics and non-diabetics were revealed. The prescription rate of diuretics was higher in patients with diabetes (80.8%) than in non-diabetics (65.1%, p<0.001). This may be due to the higher rate of patients with chronic kidney disease in diabetics and to the poorer functional status. But also the use of ß-blockers (82.7% vs. 77.5%), ACE inhibitors and angiotensin receptor blockers (80.3% vs. 77.4%) was higher in the diabetes group compared to non-diabetics.

The ATLAS trial provided information on patients with diabetes and HFrEF and the use of ACE inhibitors. In this trial low-dose and high dose lisinopril were compared [33, 34]. The results concerning the primary endpoint were similar between diabetics and non-diabetics, but the absolute benefit of high-dose lisinopril was larger in diabetics than in non-diabetics [34].

As already described by von Scheidt et al. the number of patients treated with ACEI or ARB, ß-blockers and MRA was increased from admission to discharge [23]. While the rate of ACEI or ARB use in diabetics was raised from 80.3% at admission to 87.7% at discharge the increase of ACEI or ARB treatment in non-diabetics was higher (77.4% at admission vs. 89.9% at discharge). This result may be influenced by the known protective renal effects of ACEI/ARB in diabetics.

Besides medical therapy of heart failure EVITA-HF also supplies data of non-pharmacological treatment modalities during the hospital stay. Attention should be especially paid to the use of ICDs and CRT-Ds. The rate of implanted ICDs was significantly higher in diabetics (ICD 24.0%) than in non-diabetics (ICD 21.1%, p = 0.033) at admission. As the LVEF was ≤35% in 83.8% of diabetics and 82.5% of non-diabetics and 92.6% of patients with diabetes and 86.9% of non-diabetics were in NYHA class II-IV there probably was underuse of device therapy at admission. The number of patients with implanted ICD increased from 24.0% to 30.1% in diabetics and from 21.1% to 27.3% in non-diabetics during the hospital stay. Also the number of patients carrying a CRT-D was raised from 8.1% to 14.7% in diabetics compared to an increase from 7.5% to 13.0% in non-diabetics during the hospital stay. Analyzing our data 46.0% of diabetics and 46.2% of non-diabetics had no indication for an ICD-/CRT-D implantation according to the current guidelines.

Verifying the indication for an ICD-/CRT-D implantation is important as patients with diabetes mellitus and heart failure are at increased risk of malignant ventricular arrhythmias and sudden cardiac death. For example the CHARM trial revealed a higher rate of sudden cardiac death in patients with diabetes mellitus than non-diabetics irrespective of heart failure phenotype [24, 27].

Besides the evolution in heart failure treatment there has also been progress concerning the prevention of heart failure by antidiabetic drugs. Two large randomized controlled trials investigating the cardiovascular safety of sodium-glucose co-transporter type 2 (SGLT2) inhibitors—empagliflozin and canagliflozin—have shown a significant reduction in hospitalizations because of heart failure with both drugs [35, 36]. SGLT2 inhibitors are being investigated as a potential addition to optimal medical therapy of heart failure, not only in diabetics, but also in heart failure patients without diabetes mellitus [27]. Recently the DAPA-HF trial could reveal a significant reduction of worsening heart failure or death from cardiovascular causes among patients with heart failure and reduced ejection fraction receiving dapagliflozin compared to placebo. Interestingly this effect was independent of the presence of diabetes [37]. Because of the impressive prognostic relevance the underlying pathomechanisms are currently further investigated [38, 39] to improve patient care and outcome.

As our data comprise the period between February 2009 and November 2015 we cannot assess the effect of empagliflozin and canagliflozin in our study population similar to dapagliflozin.

In contrast to other heart failure registries EVITA-HF comprises a homogenous, well-defined group of patients with chronic systolic heart failure and an ejection fraction of 40% or less [23]. EVITA-HF represents a typical cohort of heart failure patients being elderly and multi-morbid. Our data reflect the contemporary real-world management of patients with chronic heart failure with reduced ejection fraction [23]. In the present study we focused on the results of patients with diabetes and heart failure. Patients with diabetes and also non-diabetics could receive a remarkable optimization of prognosis-relevant medication during hospital stay. One of our major findings was that the mortality in patients with diabetes and DCM was significantly higher (15.2%) than in patients with DCM and no diabetes.

### Study limitations

According to the inclusion criteria of patients with chronic systolic heart failure and an ejection fraction ≤40% patients with newly diagnosed heart failure as well as patients with heart failure and a preserved ejection fraction were excluded. All participating study centers were tertiary care centers providing the full spectrum of diagnostic and treatment modalities, patients treated in other care settings were not included.

As EVITA-HF is a non-randomized registry, contribution of other covariables besides the presence of diabetes mellitus on the reported outcomes cannot fully be excluded.

In addition, further information concerning dosage of medication and the reasons for limited ICD- and CRT-use should be analyzed [23].

## Conclusions

In our study the influence of diabetes on the mortality rate was only significant in patients with dilated cardiomyopathy not in patients with ischemic heart disease. This finding suggests a distinct entity of “diabetic cardiomyopathy” independent from other pathomechanisms such as atherosclerosis.

Since undiagnosed Type 2 diabetes is common among patients with heart failure, it is prudent to screen heart failure patients without known Type 2 diabetes in accordance with current recommendations. Furthermore, as there is evidence that antidiabetic drugs like SGLT2 inhibitors have beneficial effects on patients with diabetes and heart failure further studies are required to investigate if they also have positive effects in heart failure patients without diabetes mellitus.

## Supporting information

S1 Table Medication and device status at admission and discharge in patients discharged alive. (DOCX)

S2 Table Medication at 1-year follow-up. * Documentation of follow-up interview with survivors between 300 and 450 days after index discharge.
(DOCX)

S3 Table Demographics and findings in patients with diabetes and DCM compared to diabetes and IHD at index presentation. (DOCX)

S4 Table Demographics and findings in patients with diabetes and DCM compared to diabetes and IHD at 1 year follow-up. * Documentation of follow-up interview with survivors between 300 and 450 days after index discharge. ‡ Kaplan-Meier estimates at 366 days after index discharge and p-values of the log-rank test are presented.
(DOCX)
