# Supplementary material for: The role of diabetes in cardiomyopathies of different etiologies—Characteristics and 1-year follow-up results of the EVITA-HF registry
Source: PLoS One. 2020 Jun 11;15(6):e0234260. doi: 10.1371/journal.pone.0234260 (PMC7289353; doi:10.1371/journal.pone.0234260)
Supplement: S1 Table — (DOCX) [file pone.0234260.s001.docx]

Table S1.

|  | Diabetes– admission | No diabetes– admission | p-value | Diabetes– discharge | No diabetes– discharge | p- value |
| --- | --- | --- | --- | --- | --- | --- |
| n, (%) | 1489 (36.3) | 2612 (63.7) |  | 1464 (36.2) | 2579 (63.8) |  |
| ACEI/ARB | 80.3 (1194/1487) | 77.4  (2021/2610) | 0.032 | 87.7 (1282/1462) | 89.9 (2311/2570) | 0.029 |
| ß-blocker | 82.7 (1230/1487) | 77.5 (2023/2609) | <0.001 | 92.1 (1346/1462) | 91.4 (2348/2568) | 0.48 |
| MRA | 44.2 (657/1487) | 42.3 (1104/2608) | 0.25 | 61.3 (895/1460) | 62.5 (1605/2569) | 0.46 |
| Diuretics | 80.8 (1201/1487) | 65.1 (1698/2610) | <0.001 | 89.4 (1308/1463) | 77.9 (2001/2570) | <0.001 |
| Digitalis | 23.6 (197/836) | 14.6 (210/1436) | <0.001 | 23.9 (349/1462) | 17.7 (455/2570) | <0.001 |
| ASS | 53.8 (449/835) | 42.6 (611/1435) | 0.48 | 58.1 (803/1382) | 46.4 (1134/2442) | <0.001 |
| ADP receptor inhibitors | 16.6 (139/835) | 12.3 (176/1435) | 0.004 | 23.2 (320/1382) | 32.4 (449/2442) | <0.001 |
| Oral anticoagulants | 33.3 (278/836) | 29.9 (429/1435) | 0.096 | 41.5 (606/1462) | 42.5 (1092/2569) | 0.51 |
| Statins | 64.6 (961/1487) | 48.9 (1275/2610) | <0.001 | 74.2 (1085) | 58.1 (1494/2570) | <0.001 |
| Insulin | 36.6 (304/831) | 0.7 (10/1436) | <0.001 | 39.2 (571/1456) | 0.9 (24/2568) | <0.001 |
| Oral antidiabetics | 48.5 (403/831) | 0 | <0.001 | 44.9 (654/1456) | 1.0 (26/2568) | <0.001 |
| Antidepressants | 6.8 (57/836) | 5.6 (80/1436) | 0.23 | 7.7 (112/1462) | 6.5 (167/2568) | 0.16 |
| Implanted device | 38.4 (571/1486) | 34.5 (899/2609) | 0.011 | 51.1 (747/1461) | 46.4 (1194/2576) | 0.003 |
| Pacemaker | 5.5 (81/1485) | 5.3 (137/2603) | 0.79 | 5.1 (74/1459) | 5.2 (134/2568) | 0.84 |
| ICD | 24.0 (356/1485) | 21.1 (549/2603) | 0.033 | 30.1 (439/1459) | 27.3 (700/2568) | 0.055 |
| CRT-D | 8.1 (121/1485) | 7.5 (196/2603) | 0.48 | 14.7 (214/1459) | 13.0 (334/2568) | 0.14 |
| CRT-P | 0.8 (12/1485) | 0.4 (11/2603) | 0.11 | 1.2 (18/1459) | 0.7 (18/2568) | 0.084 |
