# Supplementary material for: The role of diabetes in cardiomyopathies of different etiologies—Characteristics and 1-year follow-up results of the EVITA-HF registry
Source: PLoS One. 2020 Jun 11;15(6):e0234260. doi: 10.1371/journal.pone.0234260 (PMC7289353; doi:10.1371/journal.pone.0234260)
Supplement: S2 Table — * Documentation of follow-up interview with survivors between 300 and 450 days after index discharge. (DOCX) [file pone.0234260.s002.docx]

Table S2.

|  | Diabetes  FU | No diabetes  FU | p-value |
| --- | --- | --- | --- |
| 1-year status available, n (%)* | 928 (34.5) | 1760 (65.5) |  |
| ACEI/ARB, % (n) | 85.8 (682/795) | 87.4 (1341/1534) | 0.27 |
| ß-blocker, % (n) | 90.3 (719/796) | 88.5 (1357/1533) | 0.18 |
| MRA, % (n) | 55.6 (442/795) | 53.9 (825/1531) | 0.43 |
| Diuretics, % (n) | 83.0 (660/795) | 70.9 (1086/1532) | <0.001 |
| Digitalis, % (n) | 21.2 (169/796) | 17 (261/1537) | 0.012 |
| Amiodarone, % (n) | 6.8 (54/796) | 6.8 (105/1536) | 0.96 |

* Documentation of follow-up interview with survivors between 300 and 450 days after index discharge
