# Supplementary material for: The role of diabetes in cardiomyopathies of different etiologies—Characteristics and 1-year follow-up results of the EVITA-HF registry
Source: PLoS One. 2020 Jun 11;15(6):e0234260. doi: 10.1371/journal.pone.0234260 (PMC7289353; doi:10.1371/journal.pone.0234260)
Supplement: S3 Table — (DOCX) [file pone.0234260.s003.docx]

Table S3.

|  | DCM&diabetes | DCM&  no diabetes | p-value | ICM&diabetes | ICM&  no diabetes | p-value |
| --- | --- | --- | --- | --- | --- | --- |
| n (%) | 323 (26.7) | 885 (73.3) |  | 945 (43.3) | 1236 (56.7) |  |
| Age, yrs, median | 66 (58;74) | 59 (48;69) | <0.001 | 71 (63;77) | 71 (61;78) | 0.91 |
| Male gender, (%) n | 76.2 (246) | 72.7 (643) | 0.22 | 80.1 (757) | 82.9 (1025) | 0.09 |
| LVEF (%), median | 25 (20;32) | 27 (20;35) | 0.061 | 30 (24;35) | 30 (25;35) | 0.30 |
| Myocardial infarction, % (n) | 0 | 0 |  | 65.6 (620/945) | 65.0 (804/1236) | 0.79 |
| PCI, % (n) | 0 | 0 |  | 55.4 (502/906) | 52.0 (613/1178) | 0.13 |
| CABG, % (n) | 0 | 0 |  | 39.1 (354/906) | 30.6 (361/1178) | <0.001 |
| Art. Hypertension, % (n) | 78.9 (255/323) | 54.7 (484/885) | <0.001 | 79.6 (752/945) | 73.8 (912/1236) | 0.002 |
| Atrial fibrillation, % (n) | 40.6 (131/323) | 30.6 (271/885) | 0.001 | 35.2 (333/945) | 33.5 (414/1236) | 0.40 |
| COPD, % (n) | 18.3 (59/323) | 12.3 (109/885) | 0.008 | 15.0 (142/945) | 14.8 (183/1236) | 0.89 |
| Chronic kidney disease, % (n) | 34.1 (110/323) | 18.3 (162/884) | <0.001 | 40.7 (385/945) | 33.4 (413/1236) | <0.001 |
| Implanted device (ICD, CRT-D, CRT-P, PM), (%), (n) | 42.9 (138/322) | 32.7 (289/885) | 0.001 | 39.2 (370/944) | 39.4 (487/1235) | 0.91 |
| NYHA status III+, % (n) | 57.6 (186/323) | 47.4 (419/884) | 0.002 | 60.8 (574/944) | 54.2 (669/1235) | 0.002 |
| ACEI/ARB, % (n) | 84.8 (273/322) | 79.9 (706/884) | 0.053 | 80.5 (760/944) | 79.4 (982/1236) | 0.54 |
| ß-blocker, % (n) | 83.9 (270/322) | 79.8 (705/884) | 0.11 | 83.9 (792/944) | 79.4 (981/1235) | 0.008 |
| MRA, % (n) | 54.7 (176/322) | 49.0 (433/884) | 0.081 | 43.4 (410/944) | 41.6 (514/1235) | 0.40 |
| Diuretics | 79.5 (256/322) | 62.9 (556/884) | <0.001 | 81.5 (769/944) | 66.9 (827/1236) | <0.001 |
