# Supplementary material for: The role of diabetes in cardiomyopathies of different etiologies—Characteristics and 1-year follow-up results of the EVITA-HF registry
Source: PLoS One. 2020 Jun 11;15(6):e0234260. doi: 10.1371/journal.pone.0234260 (PMC7289353; doi:10.1371/journal.pone.0234260)
Supplement: S4 Table — * Documentation of follow-up interview with survivors between 300 and 450 days after index discharge. ‡ Kaplan-Meier estimates at 366 days after index discharge and p-values of the log-rank test are presented. (DOCX) [file pone.0234260.s004.docx]

Table S4.

|  | DCM&diabetes | DCM& no  diabetes | p-value | ICM&diabetes | ICM& no  diabetes | p-value |
| --- | --- | --- | --- | --- | --- | --- |
| One-year mortality, (%)‡ | 15.2 | 6.5 | <0.001 | 17.6 | 14.7 | 0.061 |
| MACCE, (%)‡ | 15.5 | 7.1 | <0.001 | 19.8 | 17.1 | 0.087 |
| Death, resuscitation, ICD shock, (%)‡ | 18.4 | 10.7 | <0.001 | 20.7 | 17.1 | 0.028 |
| Death or rehospitalization, (%)‡ | 45.7 | 33.6 | <0.001 | 52.8 | 49.6 | 0.12 |
| Myocardial infarction, % (n) | 0 (0/233) | 0.3 (2/704) | 0.42 | 1.7 (10/599) | 1.6 (13/838) | 0.86 |
| 1-year status available, n* | 212 | 657 |  | 579 | 790 |  |
| NYHA status III+, % (n) | 34.7 (60/173) | 23.8 (133/558) | 0.005 | 40.0 (179/447) | 31.9 (198/620) | 0.006 |
| Atrial fibrillation, % (n) | 18.9 (33/175) | 15.1 (82/544) | 0.23 | 17.5 (79/451) | 18.2 (113/620) | 0.77 |
| Chronic kidney disease, % (n) | 32.0 (64/200) | 18.7 (115/616) | <0.001 | 42.2 (226/536) | 34.4 (249/723) | 0.005 |
| Implanted device (ICD, CRT-D, CRT-P, PM), (%), (n) | 58.0 (123/212) | 50.4 (331/657) | 0.053 | 61.7 (357/579) | 56.8 (449/790) | 0.073 |
| ACEI/ARB, % (n) | 83.0 (156/188) | 90.3 (540/598) | 0.006 | 86.4 (427/494) | 85.6 (576/673) | 0.68 |
| ß-blocker, % (n) | 94.7 (178/188) | 89.3 (534/598) | 0.027 | 90.9 (450/495) | 89.9 (605/673) | 0.56 |
| MRA, % (n) | 69.7 (131/188) | 61.5 (367/597) | 0.042 | 52.8 (261/494) | 52.3 (352/673) | 0.86 |
| Diuretics, % (n) | 79.8 (150/188) | 68.2 (407/597) | 0.002 | 85.8 (424/494) | 73.3 (493/673) | <0.001 |
| Digitalis, % (n) | 28.7 (54/188) | 18.8 (113/600) | 0.004 | 18.2 (90/495) | 14.8 (100/675) | 0.12 |

* Documentation of follow-up interview with survivors between 300 and 450 days after index discharge

‡ Kaplan-Meier estimates at 366 days after index discharge and p-values of the log-rank test are presented.
